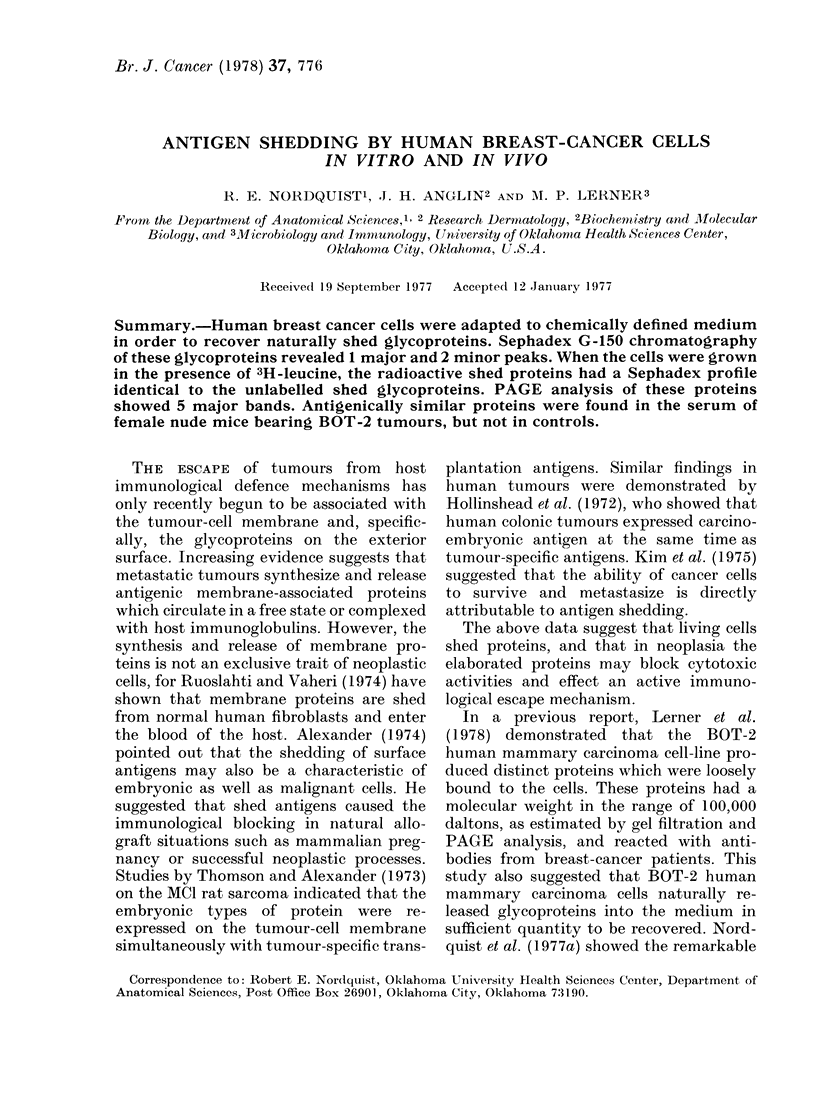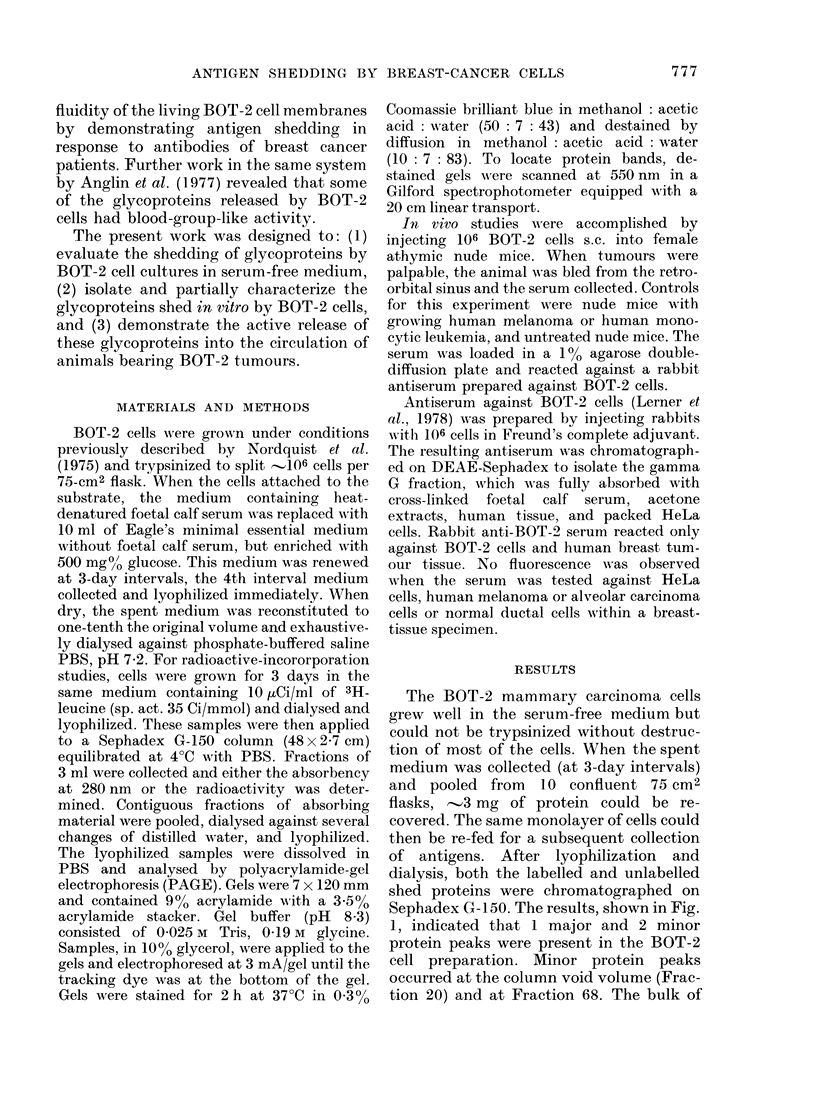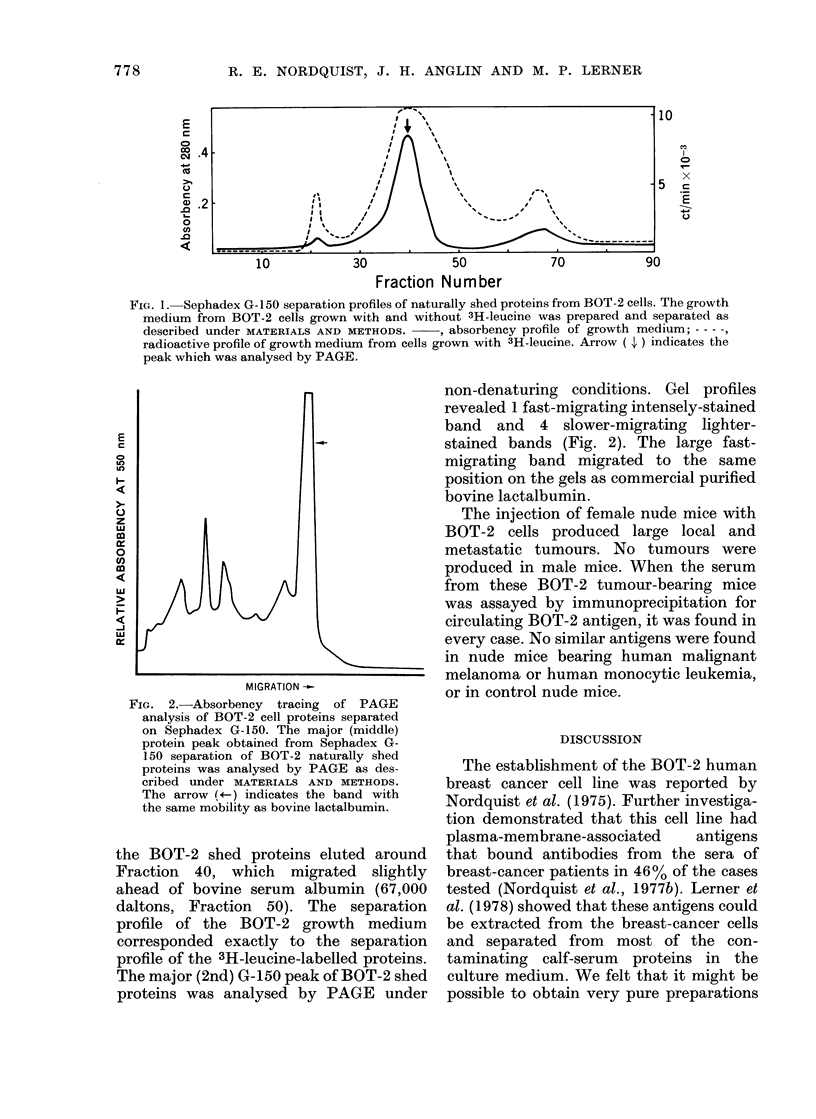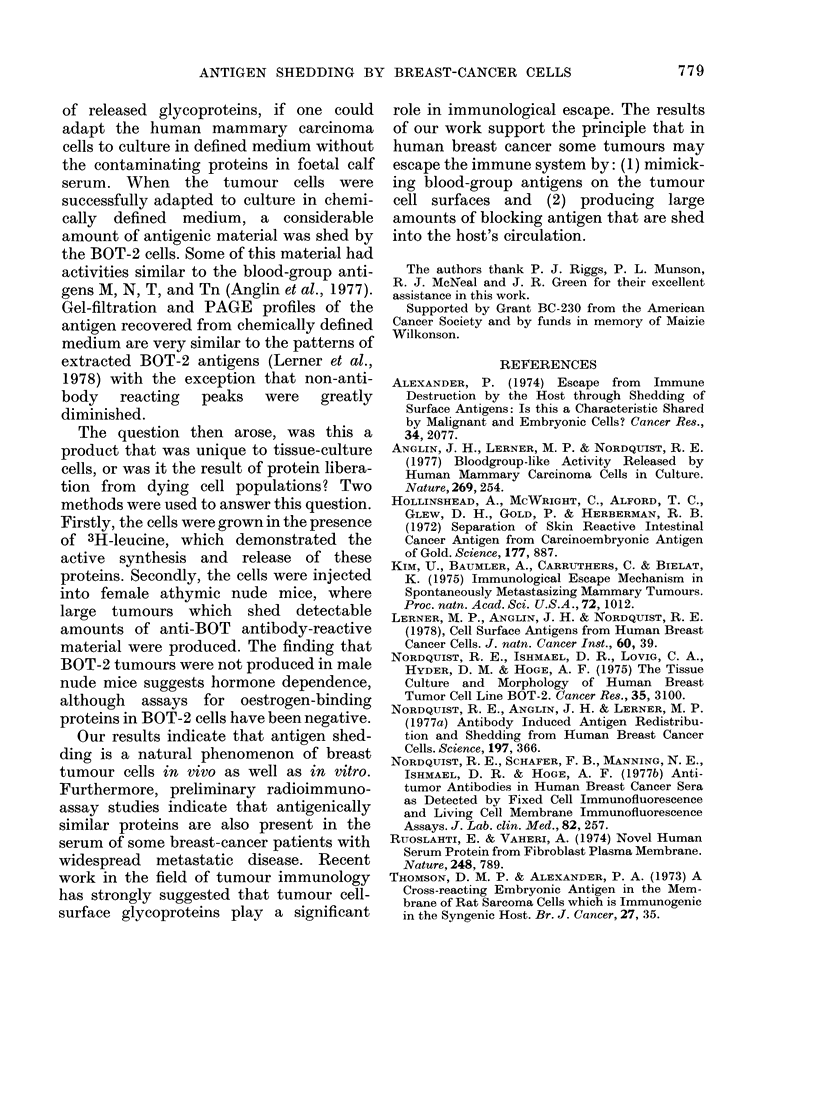# Antigen shedding by human breast-cancer cells in vitro and in vivo.

**DOI:** 10.1038/bjc.1978.115

**Published:** 1978-05

**Authors:** R. E. Nordquist, J. H. Anglin, M. P. Lerner

## Abstract

Human breast cancer cells were adapted to chemically defined medium in order to recover naturally shed glycoproteins. Sephadex G-150 chromatography of these glycoproteins revealed 1 major and 2 minor peaks. When the cells were grown in the presence of 3H-leucine, the radioactive shed proteins had a Sephadex profile identical to the unlabelled shed glycoproteins. PAGE analysis of these proteins showed 5 major bands. Antigenically similar proteins were found in the serum of female nude mice bearing BOT-2 tumours, but not in controls.


*Br. J. Cancer* (1978) **37**, 776

### ANTIGEN SHEDDING BY HUMAN BREAST-CANCER CELLS *IN VITRO* AND *IN VIVO*

R. E. NORDQUIST[1], J. H. ANGLIN[2] AND M. P. LERNER[3]

*From the Department of Anatomical Sciences,[1, 2] Research Dermatology, [2]Biochemistry and Molecular Biology, and [3]Microbiology and Immunology, University of Oklahoma Health Sciences Center, Oklahoma City, Oklahoma, U.S.A.*

Received 19 September 1977 Accepted 12 January 1977

**Summary.—Human breast cancer cells were adapted to chemically defined medium in order to recover naturally shed glycoproteins. Sephadex G-150 chromatography of these glycoproteins revealed 1 major and 2 minor peaks. When the cells were grown in the presence of $^3$H-leucine, the radioactive shed proteins had a Sephadex profile identical to the unlabelled shed glycoproteins. PAGE analysis of these proteins showed 5 major bands. Antigenically similar proteins were found in the serum of female nude mice bearing BOT-2 tumours, but not in controls.**

THE ESCAPE of tumours from host immunological defence mechanisms has only recently begun to be associated with the tumour-cell membrane and, specifically, the glycoproteins on the exterior surface. Increasing evidence suggests that metastatic tumours synthesize and release antigenic membrane-associated proteins which circulate in a free state or complexed with host immunoglobulins. However, the synthesis and release of membrane proteins is not an exclusive trait of neoplastic cells, for Ruoslahti and Vaheri (1974) have shown that membrane proteins are shed from normal human fibroblasts and enter the blood of the host. Alexander (1974) pointed out that the shedding of surface antigens may also be a characteristic of embryonic as well as malignant cells. He suggested that shed antigens caused the immunological blocking in natural allograft situations such as mammalian pregnancy or successful neoplastic processes. Studies by Thomson and Alexander (1973) on the MCl rat sarcoma indicated that the embryonic types of protein were re-expressed on the tumour-cell membrane simultaneously with tumour-specific transplantation antigens. Similar findings in human tumours were demonstrated by Hollinshead *et al.* (1972), who showed that human colonic tumours expressed carcino-embryonic antigen at the same time as tumour-specific antigens. Kim *et al.* (1975) suggested that the ability of cancer cells to survive and metastasize is directly attributable to antigen shedding.

The above data suggest that living cells shed proteins, and that in neoplasia the elaborated proteins may block cytotoxic activities and effect an active immunological escape mechanism.

In a previous report, Lerner *et al.* (1978) demonstrated that the BOT-2 human mammary carcinoma cell-line produced distinct proteins which were loosely bound to the cells. These proteins had a molecular weight in the range of 100,000 daltons, as estimated by gel filtration and PAGE analysis, and reacted with antibodies from breast-cancer patients. This study also suggested that BOT-2 human mammary carcinoma cells naturally released glycoproteins into the medium in sufficient quantity to be recovered. Nordquist *et al.* (1977*a*) showed the remarkable

Correspondence to: Robert E. Nordquist, Oklahoma University Health Sciences Center, Department of Anatomical Sciences, Post Office Box 26901, Oklahoma City, Oklahoma 73190.

fluidity of the living BOT-2 cell membranes by demonstrating antigen shedding in response to antibodies of breast cancer patients. Further work in the same system by Anglin *et al.* (1977) revealed that some of the glycoproteins released by BOT-2 cells had blood-group-like activity.

The present work was designed to: (1) evaluate the shedding of glycoproteins by BOT-2 cell cultures in serum-free medium, (2) isolate and partially characterize the glycoproteins shed *in vitro* by BOT-2 cells, and (3) demonstrate the active release of these glycoproteins into the circulation of animals bearing BOT-2 tumours.

### MATERIALS AND METHODS

BOT-2 cells were grown under conditions previously described by Nordquist *et al.* (1975) and trypsinized to split $\sim 10^6$ cells per 75-cm$^2$ flask. When the cells attached to the substrate, the medium containing heat-denatured foetal calf serum was replaced with 10 ml of Eagle's minimal essential medium without foetal calf serum, but enriched with 500 mg% glucose. This medium was renewed at 3-day intervals, the 4th interval medium collected and lyophilized immediately. When dry, the spent medium was reconstituted to one-tenth the original volume and exhaustively dialysed against phosphate-buffered saline PBS, pH 7·2. For radioactive-incororporation studies, cells were grown for 3 days in the same medium containing 10 $\mu$Ci/ml of $^3$H-leucine (sp. act. 35 Ci/mmol) and dialysed and lyophilized. These samples were then applied to a Sephadex G-150 column ($48 \times 2{\cdot}7$ cm) equilibrated at 4°C with PBS. Fractions of 3 ml were collected and either the absorbency at 280 nm or the radioactivity was determined. Contiguous fractions of absorbing material were pooled, dialysed against several changes of distilled water, and lyophilized. The lyophilized samples were dissolved in PBS and analysed by polyacrylamide-gel electrophoresis (PAGE). Gels were $7 \times 120$ mm and contained 9% acrylamide with a 3·5% acrylamide stacker. Gel buffer (pH 8·3) consisted of 0·025 M Tris, 0·19 M glycine. Samples, in 10% glycerol, were applied to the gels and electrophoresed at 3 mA/gel until the tracking dye was at the bottom of the gel. Gels were stained for 2 h at 37°C in 0·3% Coomassie brilliant blue in methanol : acetic acid : water (50 : 7 : 43) and destained by diffusion in methanol : acetic acid : water (10 : 7 : 83). To locate protein bands, destained gels were scanned at 550 nm in a Gilford spectrophotometer equipped with a 20 cm linear transport.

*In vivo* studies were accomplished by injecting $10^6$ BOT-2 cells s.c. into female athymic nude mice. When tumours were palpable, the animal was bled from the retro-orbital sinus and the serum collected. Controls for this experiment were nude mice with growing human melanoma or human monocytic leukemia, and untreated nude mice. The serum was loaded in a 1% agarose double-diffusion plate and reacted against a rabbit antiserum prepared against BOT-2 cells.

Antiserum against BOT-2 cells (Lerner *et al.*, 1978) was prepared by injecting rabbits with $10^6$ cells in Freund's complete adjuvant. The resulting antiserum was chromatographed on DEAE-Sephadex to isolate the gamma G fraction, which was fully absorbed with cross-linked foetal calf serum, acetone extracts, human tissue, and packed HeLa cells. Rabbit anti-BOT-2 serum reacted only against BOT-2 cells and human breast tumour tissue. No fluorescence was observed when the serum was tested against HeLa cells, human melanoma or alveolar carcinoma cells or normal ductal cells within a breast-tissue specimen.

### RESULTS

The BOT-2 mammary carcinoma cells grew well in the serum-free medium but could not be trypsinized without destruction of most of the cells. When the spent medium was collected (at 3-day intervals) and pooled from 10 confluent 75 cm$^2$ flasks, $\sim$3 mg of protein could be recovered. The same monolayer of cells could then be re-fed for a subsequent collection of antigens. After lyophilization and dialysis, both the labelled and unlabelled shed proteins were chromatographed on Sephadex G-150. The results, shown in Fig. 1, indicated that 1 major and 2 minor protein peaks were present in the BOT-2 cell preparation. Minor protein peaks occurred at the column void volume (Fraction 20) and at Fraction 68. The bulk of

FIG. 1.—Sephadex G-150 separation profiles of naturally shed proteins from BOT-2 cells. The growth medium from BOT-2 cells grown with and without $^3$H-leucine was prepared and separated as described under MATERIALS AND METHODS. ——, absorbency profile of growth medium; - - - -, radioactive profile of growth medium from cells grown with $^3$H-leucine. Arrow ( ↓ ) indicates the peak which was analysed by PAGE.

FIG. 2.—Absorbency tracing of PAGE analysis of BOT-2 cell proteins separated on Sephadex G-150. The major (middle) protein peak obtained from Sephadex G-150 separation of BOT-2 naturally shed proteins was analysed by PAGE as described under MATERIALS AND METHODS. The arrow (←) indicates the band with the same mobility as bovine lactalbumin.

the BOT-2 shed proteins eluted around Fraction 40, which migrated slightly ahead of bovine serum albumin (67,000 daltons, Fraction 50). The separation profile of the BOT-2 growth medium corresponded exactly to the separation profile of the $^3$H-leucine-labelled proteins. The major (2nd) G-150 peak of BOT-2 shed proteins was analysed by PAGE under non-denaturing conditions. Gel profiles revealed 1 fast-migrating intensely-stained band and 4 slower-migrating lighter-stained bands (Fig. 2). The large fast-migrating band migrated to the same position on the gels as commercial purified bovine lactalbumin.

The injection of female nude mice with BOT-2 cells produced large local and metastatic tumours. No tumours were produced in male mice. When the serum from these BOT-2 tumour-bearing mice was assayed by immunoprecipitation for circulating BOT-2 antigen, it was found in every case. No similar antigens were found in nude mice bearing human malignant melanoma or human monocytic leukemia, or in control nude mice.

### DISCUSSION

The establishment of the BOT-2 human breast cancer cell line was reported by Nordquist *et al.* (1975). Further investigation demonstrated that this cell line had plasma-membrane-associated antigens that bound antibodies from the sera of breast-cancer patients in 46% of the cases tested (Nordquist *et al.*, 1977*b*). Lerner *et al.* (1978) showed that these antigens could be extracted from the breast-cancer cells and separated from most of the contaminating calf-serum proteins in the culture medium. We felt that it might be possible to obtain very pure preparations of released glycoproteins, if one could adapt the human mammary carcinoma cells to culture in defined medium without the contaminating proteins in foetal calf serum. When the tumour cells were successfully adapted to culture in chemically defined medium, a considerable amount of antigenic material was shed by the BOT-2 cells. Some of this material had activities similar to the blood-group antigens M, N, T, and Tn (Anglin *et al.*, 1977). Gel-filtration and PAGE profiles of the antigen recovered from chemically defined medium are very similar to the patterns of extracted BOT-2 antigens (Lerner *et al.*, 1978) with the exception that non-antibody reacting peaks were greatly diminished.

The question then arose, was this a product that was unique to tissue-culture cells, or was it the result of protein liberation from dying cell populations? Two methods were used to answer this question. Firstly, the cells were grown in the presence of $^3$H-leucine, which demonstrated the active synthesis and release of these proteins. Secondly, the cells were injected into female athymic nude mice, where large tumours which shed detectable amounts of anti-BOT antibody-reactive material were produced. The finding that BOT-2 tumours were not produced in male nude mice suggests hormone dependence, although assays for oestrogen-binding proteins in BOT-2 cells have been negative.

Our results indicate that antigen shedding is a natural phenomenon of breast tumour cells *in vivo* as well as *in vitro*. Furthermore, preliminary radioimmunoassay studies indicate that antigenically similar proteins are also present in the serum of some breast-cancer patients with widespread metastatic disease. Recent work in the field of tumour immunology has strongly suggested that tumour cell-surface glycoproteins play a significant role in immunological escape. The results of our work support the principle that in human breast cancer some tumours may escape the immune system by: (1) mimicking blood-group antigens on the tumour cell surfaces and (2) producing large amounts of blocking antigen that are shed into the host's circulation.

The authors thank P. J. Riggs, P. L. Munson, R. J. McNeal and J. R. Green for their excellent assistance in this work.

Supported by Grant BC-230 from the American Cancer Society and by funds in memory of Maizie Wilkonson.

### REFERENCES

ALEXANDER, P. (1974) Escape from Immune Destruction by the Host through Shedding of Surface Antigens: Is this a Characteristic Shared by Malignant and Embryonic Cells? *Cancer Res.*, **34**, 2077.

ANGLIN, J. H., LERNER, M. P. & NORDQUIST, R. E. (1977) Bloodgroup-like Activity Released by Human Mammary Carcinoma Cells in Culture. *Nature*, **269**, 254.

HOLLINSHEAD, A., MCWRIGHT, C., ALFORD, T. C., GLEW, D. H., GOLD, P. & HERBERMAN, R. B. (1972) Separation of Skin Reactive Intestinal Cancer Antigen from Carcinoembryonic Antigen of Gold. *Science*, **177**, 887.

KIM, U., BAUMLER, A., CARRUTHERS, C. & BIELAT, K. (1975) Immunological Escape Mechanism in Spontaneously Metastasizing Mammary Tumours. *Proc. natn. Acad. Sci. U.S.A.*, **72**, 1012.

LERNER, M. P., ANGLIN, J. H. & NORDQUIST, R. E. (1978), Cell Surface Antigens from Human Breast Cancer Cells. *J. natn. Cancer Inst.*, **60**, 39.

NORDQUIST, R. E., ISHMAEL, D. R., LOVIG, C. A., HYDER, D. M. & HOGE, A. F. (1975) The Tissue Culture and Morphology of Human Breast Tumor Cell Line BOT-2. *Cancer Res.*, **35**, 3100.

NORDQUIST, R. E., ANGLIN, J. H. & LERNER, M. P. (1977*a*) Antibody Induced Antigen Redistribution and Shedding from Human Breast Cancer Cells. *Science*, **197**, 366.

NORDQUIST, R. E., SCHAFER, F. B., MANNING, N. E., ISHMAEL, D. R. & HOGE, A. F. (1977*b*) Antitumor Antibodies in Human Breast Cancer Sera as Detected by Fixed Cell Immunofluorescence and Living Cell Membrane Immunofluorescence Assays. *J. Lab. clin. Med.*, **82**, 257.

RUOSLAHTI, E. & VAHERI, A. (1974) Novel Human Serum Protein from Fibroblast Plasma Membrane. *Nature*, **248**, 789.

THOMSON, D. M. P. & ALEXANDER, P. A. (1973) A Cross-reacting Embryonic Antigen in the Membrane of Rat Sarcoma Cells which is Immunogenic in the Syngenic Host. *Br. J. Cancer*, **27**, 35.